# Protection against Congenital CMV Infection Conferred by MVA-Vectored Subunit Vaccines Extends to a Second Pregnancy after Maternal Challenge with a Heterologous, Novel Strain Variant

**DOI:** 10.3390/v13122551

**Published:** 2021-12-20

**Authors:** Claudia Fernández-Alarcón, Grace Buchholz, Heidi Contreras, Felix Wussow, Jenny Nguyen, Don J. Diamond, Mark R. Schleiss

**Affiliations:** 1 Division of Pediatric Infectious Diseases, Department of Pediatrics, University of Minnesota Medical School, Minneapolis, MN 55455, USA; ferna128@umn.edu (C.F.-A.); buchh123@umn.edu (G.B.); 2 Department of Hematology and Transplant Center, City of Hope National Medical Center, Duarte, CA 91010, USA; hcontreras@coh.org (H.C.); fussow@coh.org (F.W.); jenguyen@coh.org (J.N.); DDiamond@coh.org (D.J.D.)

**Keywords:** CMV vaccine, CMV reinfection, CIDTMR strain, TAMYC, pentameric complex, gO, congenital CMV, guinea pig

## Abstract

Maternal reinfection of immune women with novel human cytomegalovirus (HCMV) strains acquired during pregnancy can result in symptomatic congenital CMV (cCMV) infection. Novel animal model strategies are needed to explore vaccine-mediated protections against maternal reinfection. To investigate this in the guinea pig cytomegalovirus (GPCMV) model, a strictly in vivo-passaged workpool of a novel strain, the CIDMTR strain (dose, 1 × 10^7^ pfu) was used to infect dams that had been challenged in a previous pregnancy with the 22122 strain, following either sham-immunization (vector only) or vaccination with MVA-vectored gB, gH/gL, or pentameric complex (PC) vaccines. Maternal DNAemia cleared by day 21 in the glycoprotein-vaccinated dams, but not in the sham-immunized dams. Mean pup birth weights were 72.85 ± 10.2, 80.0 ± 6.9, 81.4 ± 14.1, and 89.38 ± 8.4 g in sham-immunized, gB, gH/gL, and PC groups, respectively (*p* < 0.01 for control v. PC). Pup mortality in the sham-immunized group was 6/12 (50%), but reduced to 3/35 (8.6%) in combined vaccine groups (*p* = 0.0048). Vertical CIDMTR transmission occurred in 6/12 pups (50%) in the sham-vaccinated group, compared to 2/34 pups (6%) in the vaccine groups (*p* = 0.002). We conclude that guinea pigs immunized with vectored vaccines expressing 22122 strain-specific glycoproteins are protected after a reinfection with a novel, heterologous clinical isolate (CIDMTR) in a second pregnancy.

## 1. Introduction

An enigmatic but critical concept in HCMV epidemiology that is relevant to pregnancy outcomes and to vaccine-mediated prevention against congenital CMV (cCMV) infection is the phenomenon of maternal reinfection. In contrast to other viral diseases associated with maternal–fetal transmission, such as rubella and Zika virus infection, the prevalence of cCMV infection is directly, and not inversely, proportional to the overall prevalence of CMV antibodies in a given population of women of child-bearing age [1]. Although pre-conception seropositivity probably provides some benefits, including a reduction of the clinical severity of cCMV if transmission occurs [2], it does not provide full protection against maternal reinfection. Reinfections in turn can be accompanied by fetal transmission during pregnancy [3]. Such transmissions can lead to symptomatic cCMV disease, including sensorineural hearing loss [4]. Although some cCMV infections may occur as a result of reactivation of a latent infection, the biggest burden engendered by cCMV infection appears to be in infants born to women with pre-conception immunity [5,6,7,8] who are then reinfected with heterologous strain variants during their subsequent pregnancies. In fact, it is estimated that the majority of cases of cCMV infection are due to maternal reinfections during pregnancy [3].

Several factors appear to contribute to reinfections with new, novel strains of CMV in seropositive hosts. One reason that convalescent immunity to HCMV is incomplete, even under the best of circumstances, is because of the abundance of virally encoded immune evasion genes that interfere with the clearance of infection [9]. Indeed, HCMV undergoes extensive intergenic and intragenic recombination, generating an extraordinarily diverse set of strain variants [10,11,12]. Reinfection with variant strains, particularly strains with polymorphisms in epitopes in immunodominant envelope glycoproteins, appears to be common [13]. It has been estimated that approximately 75% of congenital CMV infections in the U.S., and an even higher percentage globally, occur in the setting of recurrent maternal infection during pregnancy [14]. It is controversial whether maternal pre-existing seroimmunity substantially reduces the risk for neurodevelopmental sequelae in the newborn infant if transmission of HCMV occurs [3]. Sequelae, including sensorineural hearing loss (SNHL), have been demonstrated in numerous studies of congenitally infected infants born to women with preconception immunity [6,7,8,15], and viral strain variation appears to play a key role in such transmissions.

Against this backdrop, how can a vaccine against cCMV–based on immunogens corresponding to a single strain of a virus–provide protection for the pregnant patient against all strains that may be encountered, and how can we examine these questions in animal models of cCMV transmission? Efforts to use animal models to study the impact of strain variation on reinfection have yielded minimal data to date. Laboratory-adapted and wild-type strains of murine cytomegalovirus (MCMV) demonstrated significant sequence variation, particularly at the genomic termini [16], but MCMV is not vertically transmitted, making it impossible to perform studies of the impact of strain variation in MCMV-naïve or immune mice on congenital infection. In contrast, variants of rhesus macaque CMV (rhCMV), such as rhCMV UCD59, 180.92, and UCD52, have been used to challenge pregnant macaques [17], making this a tractable model to study the impact of strain variation on cCMV transmission. Although both of these species-specific CMVs have also been useful for the study of vaccines [18,19], neither the MCMV nor the rhCMV model have been used to examine the protective impact of vaccine-acquired immunity initially engendered against a reference strain (either from natural infection or vaccination) of virus, followed by reinfection with a divergent, heterologous strain.

Toward the goal of modeling reinfection in the GPCMV model, we recently isolated a novel viral strain, designated CIDMTR, through an allogeneic explant reactivation strategy using salivary gland (SG) homogenates from guinea pigs that were identified in a commercial animal colony [20,21]. The sequence of the 232,778-nucleotide CIDMTR genome (accession #HG531783) was mostly well-conserved with that of the 22122 reference strain. The overall sequence homology to strain 22122 (accession #KC503762.1) was 98%, although examination of some regions of sequence divergence revealed CIDMTR-strain-specific open reading frames, including a second copy of a gene product predicted for the gp147 gene family, annotated as gene *gp147.1*, which encodes a putative MHC-1 homolog [22]. Previous work had demonstrated that GPCMV-seropositive female guinea pigs (infected with the 22122 strain) were, after establishment of pregnancy, susceptible to reinfection with CIDMTR, and that fetal transmission of the heterologous variant occurred in 22122-seropositive dams. In a previously reported study of twelve 22122-seropositive dams, we evaluated for vertical transmission to the fetus after heterologous challenge with the CIDMTR strain. Six dams were sham-challenged, and 6 were challenged with CIDMTR virus. We noted that over 20% of liveborn pups in the challenge group had evidence of congenital transmission of CIDMTR, as evidenced by strain-specific PCR [21]; in contrast, the 22122 seropositive dams that were sham-inoculated demonstrated no evidence of cCMV (with either the 22122 or CIDMTR sets of strain-specific primers) in any pup. Given that natural GPCMV-seropositivity to the 22122 strain was insufficient to protect against cCMV transmission due to the heterologous CIDMTR strain in this initial study, in the current report we evaluated whether the 22122-specific immunity imparted by MVA-vectored glycoprotein subunit vaccines (gB; gH/gL; and the GPCMV PC of gH/gL/GP129/131/133) was sufficient to protect against heterologous reinfection and congenital transmission with the novel CIDMTR strain. Since CIDMTR demonstrates substantial differences in glycoprotein-coding content compared to the 22122 sequences used in the generation of the MVA vaccines administered prior to the first pregnancy [23], these studies provided a translationally relevant evaluation of the “cross-strain” potential for protection for vaccine-acquired immunity that could be achieved in a second pregnancy when a new, novel strain of CMV was encountered by the pregnant host.

## 2. Materials and Methods

### 2.1. Guinea Pigs

Outbred Hartley guinea pigs were purchased from Elm Hill Laboratories (Chelmsford, MA, USA). Strain 2 guinea pigs (for propagation of SG stocks) were maintained in the University of Minnesota (UMN) vivarium. The CIDMTR viral stock was originally isolated from GPCMV-seropositive animals commercially purchased from Charles River Laboratories [21]. All animals used prior to commencing these vaccine/challenge studies were confirmed to be GPCMV-seronegative by ELISA and housed under conditions approved by the Institutional Animal Care and Use Committee (IACUC) at the UMN (protocol 1607-33994A, originally approved in 2016, and renewed by the IACUC in 2019).

### 2.2. Cells and Virus

**Figure A1:**
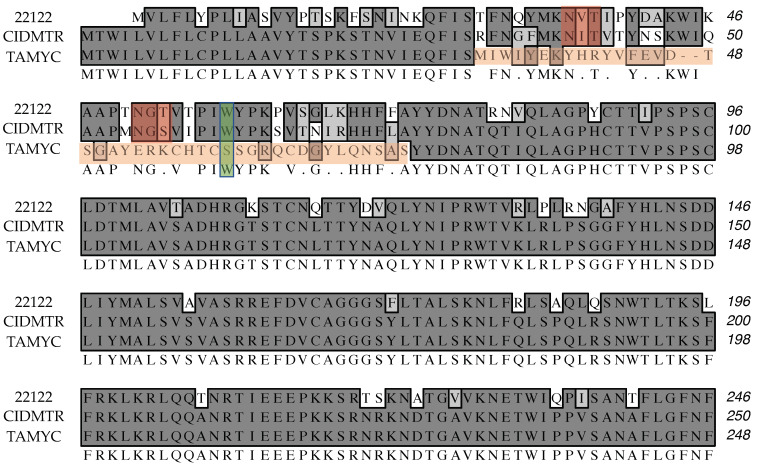
GPCMV strains 22122, CIDMTR, and TAMYC. (**a**) Sequence of CIDMTR strain noted to be identical to TAMYC except for 46 aa region (orange shading) with no known or identifiable homology to any herpesvirus sequence on GenBank database. Within this region, a 15 aa sequence CSSGRQCDGYLQNSA was noted to have identity to prokaryotic nitrogenase reductase ORF (details in text). Missing n-linked glycosylation sites in TAMYC ORF are as outlined in red shading. A highly conserved tryptophan residue in the UL74 protein family in all betaherpeviruses [33] is highlighted in green.

Cell culture for GPCMV was carried out in guinea pig lung fibroblast cells (GPL; ATCC CCL158) in F-12 medium, supplemented with 10% fetal calf serum (FCS, ThermoFisher Scientific, Waltham, MA, USA) 10,000 IU/L penicillin, 10 mg/L streptomycin (Gibco-BRL) and 0.75% NaHCO_3_ (Gibco-BRL), as previously described [23]. Viral stocks of CIDMTR strain were maintained by passaging the original SG homogenate in vivo in strain 2 animals; only viruses that had been exclusively passaged as organ homogenate in vivo, with no intermediate tissue culture passage or cultivation step, were used for animal challenge studies. To confirm the identity of the CIDMTR challenge virus, PCR and Sanger sequencing of the relevant ORFs (Table A1) was performed, confirming the previously reported sequences in the CIDMTR virus [21]. Comparisons were also performed for the glycoprotein O (gO) ORF as a confirmation of the identity of the CIDMTR challenge virus, since this ORF is the most hypervariable of the glycoprotein coding sequences (Figure A1).

### 2.3. Generation of Vaccine Constructs, Experimental Design, and Pregnancy Challenge

The construction of the MVA-vectored vaccines has been previously described [23]. Modified vaccinia virus Ankara (MVA) vectors, expressing gB, gH/gL, or the GPCMV PC subunits, were engineered. A total of eight animals/group were subcutaneously administered a three-dose series of vaccines (3 × 10^7^ pfu/dose). Guinea pig breeding was commenced within 14 days after the third vaccination. Pregnancies were monitored by palpation, and dams were challenged in the early third trimester with SG-adapted GPCMV, strain 22122 [24], at a dose of 1 × 10^5^ PFU, by subcutaneous route. Pregnancy outcomes (maternal viremia, birth weights, live/dead pups, and congenital infection rates) were then monitored. All live-born pups were sacrificed within 72 h of delivery for organ harvest and PCR analysis, with comparisons made to viral load in the visceral organs of stillborn pups, harvested at the time of delivery. Results of this study were previously reported [23].

To assess the comparative effects of pre-conception vaccination on pregnancy outcomes following an experimental heterologous GPCMV infection during a second pregnancy, in animals immunized with the 22122-specific MVA vaccines, a pregnancy/challenge study was conducted. A subset of animals from the original 22122 challenge study were placed with seronegative breeders and a second pregnancy established. An in vivo-passaged CIDMTR workpool (1 × 10^7^ pfu) was used to infect dams, by subcutaneous (SC) route, during the mid-second trimester. As noted, these dams had previously challenged in a prior (first) pregnancy with SG-adapted 22122. In this reinfection experiment, they were challenged with tissue-homogenate in vivo passaged CIDMTR virus. Animals used in this reinfection experiment were from the sham (vector) immunization (*n* = 3) group, or the MVA-vectored gB (*n* = 2), gH/gL (*n* = 3), or PC (*n* = 4) vaccine groups, respectively.

Maternal blood was obtained on 7, 14, and 21 days post-challenge with in vivo-passaged CIDMTR virus, and analyzed for viral load by qPCR, as described previously [23]. Briefly, DNA was extracted from either 100 μL citrated maternal blood, or from pup tissues, using 0.05 g of homogenized frozen samples of liver, lung, or spleen (QIAamp 96 DNA QIAcube HT Kit, Qiagen, Hilden, Germany). Amplification primers employed were as previously described for the CIDMTR strain [21], to confirm that reinfection had occurred with the heterologous (reinfecting) CIDMTR strain, and was not due to reactivation of the original 22122 strain used for the previous challenge. These PCR primers allowed differentiation of CIDMTR-specific sequence, compared to strain 22122 sequence, focused on the amplification of sequences corresponding to the CIDMTR strain gp147.1 ORF, absent from the 22122 strain. A GPCMV gp147.1 ORF-specific real-time PCR primer pair, consisting of CIDMTR147.1_464F (5′-ATGCAACATAGCGTGCTGAC-3′) and CIDMTR147.1_583R (5′-GGGACAAAAGCACGATGAAC-3′) was designed and utilized. These primers amplified a 120 bp region of the *gp147.1* gene specific for the CIDMTR strain. The specific hydrolysis probe used for detection was CIDMTR147.1_494P (FAM-GTGTTCGTGTCCTTGATCGTACGCA-BHQ1).

Data were analyzed with the LightCycler Data Analysis Software (version 1.5; Roche). DNAemia was expressed as the total number of genome copies per mL of blood (limit of detection ~200 copies/mL). A level of 100 copies/mL was assigned to negative blood samples, and one copy/mg to negative tissue samples. Tissue viral loads were expressed as genome copies per mg of tissue. The limit of detection for the PCR assay was two genome copies/mg tissue.

### 2.4. Statistical Analyses

GraphPad Prism (version 8.0, San Diego, CA, USA) was used for statistical analyses. Pup mortality and transmission data were compared using Fisher’s exact test with one-sided comparisons. Antibody titers and visceral viral loads were compared using non-parametric comparisons (Mann–Whitney and Kruskal–Wallis). Parametric data (pup birth weights) were compared using two-way analysis of variance (ANOVA), with values adjusted by Bonferroni’s multiple-comparison method.

## 3. Results

### 3.1. Dams Previously Immunized with 22122 Strain-Specific MVA Vaccines Were Challenged with 22122 GPCMV in Their First Pregnancies

We previously reported the impact of pre-conception vaccination with MVA-vectored vaccines expressing GPCMV gB, gH/gL, and the PC on pregnancy outcomes following an early third trimester challenge with SG-adapted 22122 [23], the prototypical strain of GPCMV [25]. Pre-conception vaccination was performed using MVA expression constructs based on the 22122 ORF sequences, prior to homologous challenge with the 22122 virus. All vaccines elicited GPCMV-specific binding and neutralizing antibodies, and pre-conception immunization resulted in reductions ranging from 4.9- to 698-fold in maternal DNAemia following the SG virus challenge in the first pregnancy. Maternal vaccination with the MVA vaccines also improved the pups’ birth weights, reduced mortality, and, for MVA-gB, reduced transmission. In the current study, we extended these observations to examine whether previous immunization against the 22122 strain-specific sequences conferred protection extending into a second pregnancy, following a mid-second trimester challenge with a heterologous strain of GPCMV, the CIDMTR strain [20,21].

For establishment of a second pregnancy, guinea pigs remaining from the previous pregnancy/challenge study were used. Some animals from the previous study had been necropsied or euthanized. In total, twelve animals were available for mating to establish a second pregnancy and were used for these studies (Table 1). Notably, animals were available from each of the four experimental groups previously reported [23]. Dams that were used for this heterologous reinfection study were from the sham (Venus vector only) group (*n* = 3); the MVA-vectored gB group (*n* = 2); the gH/gL group (*n* = 3); and the PC (*n*-4) group. In the previous challenge study [23], the pup mortality and vertical transmission rates in the Venus control group were both 100% (Table 1), whereas there was no pup mortality or cCMV transmission in any dam from the vaccine groups for those animals available for mating, establishment of a second pregnancy, and heterologous challenge with a novel strain of GPCMV. Only the in vivo-passaged CIDMTR virus was used for pregnancy challenge, to avoid any complicating variables that might be associated with the development of mutations in genomic sequence following cell culture passage. Post-immunization geometric mean ELISA titers, prior to establishment of a second pregnancy, were: Venus (control), 40 ± standard deviation factor (SDF) 1.0; gB, 3620 ± 4.4; gH/gL 2560 ± 1.0; PC, 1522 ± 1.4. Animals were placed with GPCMV-seronegative breeder males and the second pregnancies monitored as previously described [24]. The ELISA, western blot, and neutralization titer results induced by MVA vaccines were previously published [23].

### 3.2. Previous Immunization with 22122 Subunit MVA Vaccines Reduces Maternal DNAemia Following Second Trimester Heterologous CIDMTR Challenge in a Second Pregnancy

**Figure 1:**
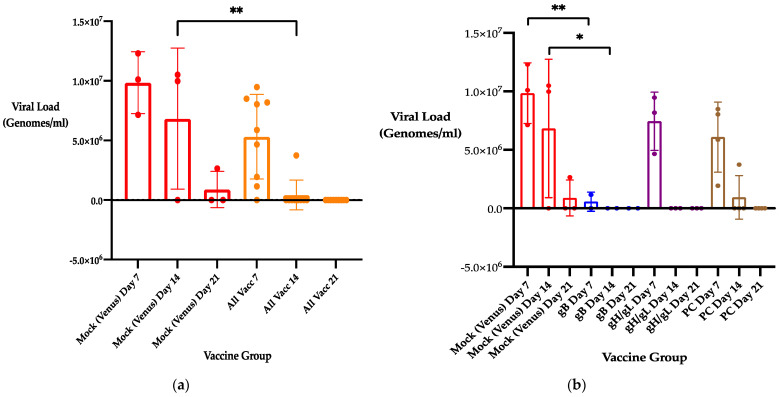
Scatter plots (with bars) comparing maternal DNAemia in second pregnancy following heterologous CIDMTR infectious challenges in dams that have been mock-immunized, or immunized with MVA-vectored GPCMV glycoprotein constructs [23]. Data shown are mean viral loads ± SD: (**a**) Comparison of Venus-vectored vaccination (red bars and scatter plots) with pooled data for all glycoprotein vaccines (orange scatter plots/bars) for all three groups. Across all vaccine groups, reductions in viral load were noted (*p* < 0.01 for day 14 time point). (**b**) Comparison of vaccination for each individual construct (Venus [red]; gB [blue]; gH/gL [purple]; and PC [brown]). Significant difference noted by ANOVA for gB vaccination compared to Venus control for viral loads at day 7 (*p* < 0.01) and day 14 (*p* < 0.05). *, *p* < 0.05; **, *p* < 0.01.

To assess the impact of previous immunization with MVA-vectored GPCMV subunit vaccines upon maternal DNAemia following challenge with a heterologous strain of GPCMV (CIDMTR) during a subsequent pregnancy, pregnant dams (as described in Section 3.1) were challenged with CIDMTR virus in the mid-second trimester. Care was given to perform SC challenge only with tissue homogenate of a serially passaged CIDMTR virus that had never been passaged in cell culture. Next, we examined for maternal DNAemia at days 7, 14, and 21 post-CIDMTR challenge in challenged animals. In animals previously sham-immunized with MVA vector only (Venus group), maternal DNAemia persisted at high levels through 21 days post-challenge (7.0 ± 0.1, 5.8 ± 2.1, and 3.5 ± 2.6 log_10_ genome copies [±SD]/mL at days 7, 14, and 21, respectively (Figure 1). In dams immunized from all vaccine groups combined, maternal DNAemia levels were 6.2 ± 1.6, 2.5 ± 1.5, and 2.0 ± 0 (limit of assay detection) log_10_ genome copies/mL at days 7, 14 and 21 post-challenge, respectively (Figure 1a; these levels were significant at *p* < 0.01 for mock (Venus) vaccine at day 14, versus the combined vaccine groups).

When vaccine groups were considered individually (Figure 1b), for the MVA/gB group, viral load was 4.0 ± 2.9 log_10_ genome copies/mL at day 7, and 2.0 ± 0 log_10_ genome copies/mL (threshold of detection) at days 14 and 21. In dams immunized with MVA/gH/gL, viral load was 6.9 ± 0.16 log_10_ genome copies/mL at day 7, and 2.0 ± 0 log_10_ genome copies/mL (threshold of detection) at days 14 and 21. In dams immunized with MVA/PC vaccine, viral load was 6.7 ± 0.3 log_10_ genome copies/mL at day 7, 3.1 ± 2.3 log_10_ genome copies/mL at day 14, and 2.0 ± 0 log_10_ genome copies/mL (threshold of detection) at day 21. By ANOVA comparing the Venus and gB groups, these differences were noted to be statistically significant at both day 7 (*p* < 0.01) and at day 14 (*p* < 0.05; Figure 1b).

### 3.3. Previous Immunization with 22122 Subunit MVA Vaccines Improves Pregnancy Outcomes (Pup Weights and Pup Survival) following Heterologous Challenge with CIDMTR Virus

We next examined whether the duration of pregnancy was impacted by a past history of immunization. Mock (Venus)-immunized dams had a mean duration of pregnancy post-CIDMTR challenge of 20.7 ± 8 (SD). The mean duration of pregnancy post-CIDMTR virus challenge was 28 ± 1.4 days in the gB group; 23.7 ± 2.9 in the gH/gL group; and 28.8 ± 8.8 in the PC group (P = NS).

The impact of vaccination on pup survival after challenge during pregnancy with CIDMTR was compared. A total of 12 pups were born to the Venus (vector only) group, with a pup mortality rate of 6/12 (50%). Among all three vaccine groups, a total of 35 pups were born, with a pup mortality rate of 3/35 (8.6%). This was a highly significant difference (*p* = 0.0048, Fisher’s exact test) in mortality between the Venus control and the combined vaccine groups. The breakdown in mortality per group is noted in Table 2.

**Figure 2:**
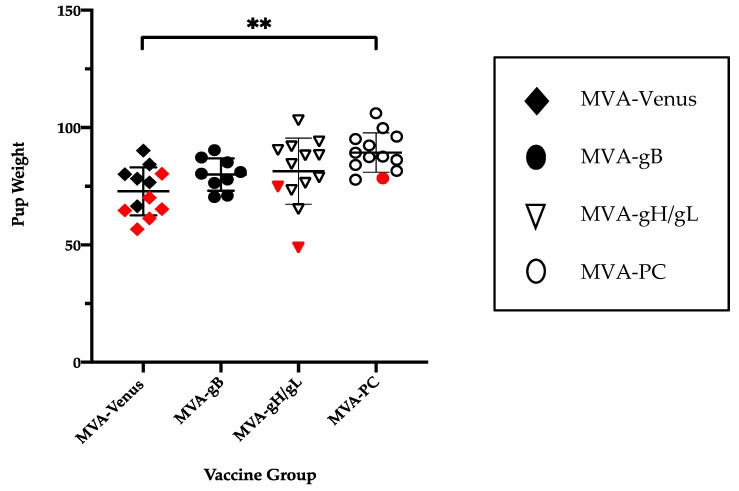
Scatter plots of pup weights in litters following maternal reinfection. (**a**) Mean pup birth weights, expressed as mean ± SD. Mean weights shown for sham-immunized, gB, gH/gL, and PC groups, respectively (*p* < 0.01 for MVA-PC versus MVA-Venus). Red symbols designate a dead (stillborn) pup. The newborn weights were statistically significantly improved in pups born to dams previously immunized with MVA-PC vaccine (*p* < 0.01). **, *p* < 0.01.

Newborn pup weights were compared in the vaccine groups (Figure 2). Mean pup birth weight was 72.85 ± 10.2, 80.0 ± 6.9, 81.4 ± 14.1, and 89.38 ± 8.4 g in sham-immunized, gB, gH/gL, and PC groups, respectively. Pup weights trended higher in vaccine groups, but were significantly higher in the pups born to the MVA/PC group (*p* < 0.01).

### 3.4. Previous Immunization with 22122-based Subunit MVA Vaccines Reduces Congenital GPCMV Transmission and Pup Viral Load from a Heterologous Strain following Viral Challenge in a Subsequent Pregnancy

**Figure 3:**
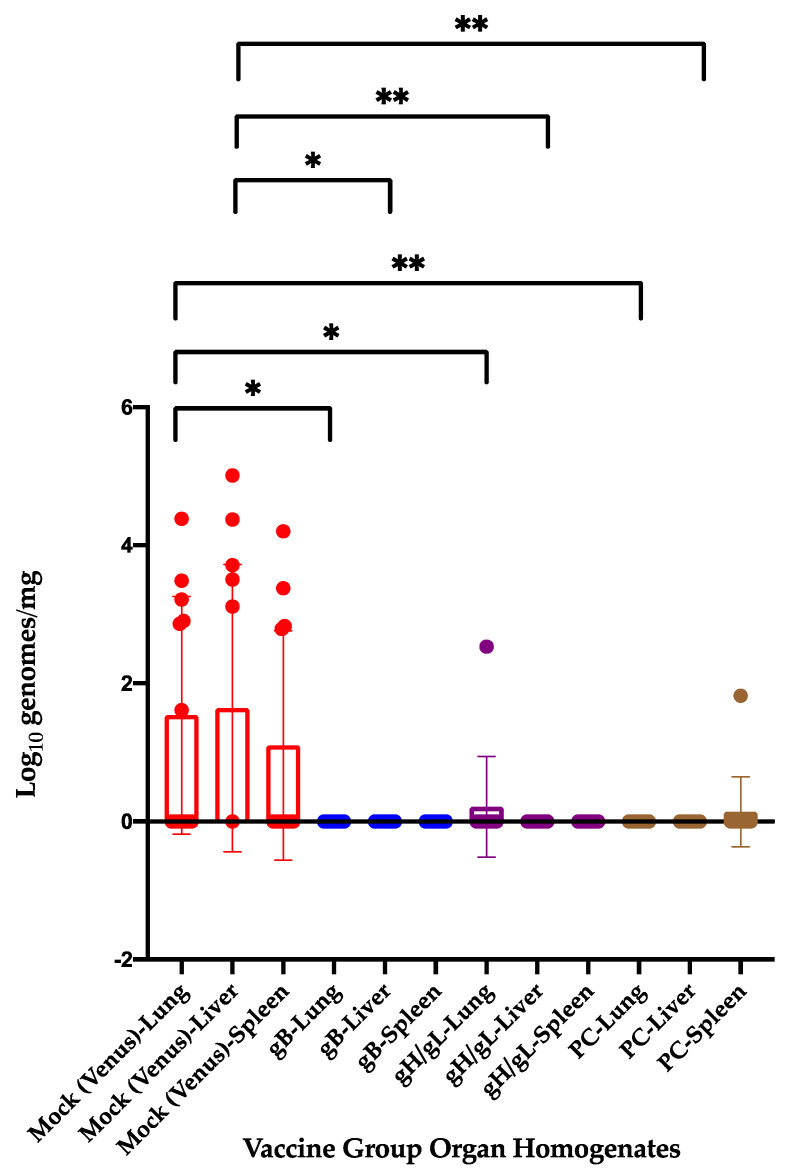
Scatter plot with bars evaluating impact of MVA vaccination on visceral organ viral loads in pups and congenital GPCMV transmission from CIDMTR strain. PCR performed with CIDMTR-specific primers on organ homogenates of lung, liver, and spleen from pups born to Venus-vaccinated and glycoprotein-vaccinated dams. Significant differences were observed in viral load in lung tissue from the Venus group versus the gB group (*p* < 0.05) and versus the gH/gL and PC groups (*p* < 0.01). For liver, similar differences were noted; gH/gL group (*p* < 0.01); and PC group (*p* < 0.05). Significant reductions in liver viral load were also noted, comparing Venus pups with gB (*p* < 0.05); gH/gL (*p* < 0.01); and PC (*p* < 0.01). Color code: Venus [red]; gB [blue]; gH/gL [purple]; and PC [brown]. *, *p* < 0.05; **, *p* < 0.01.

Finally, we examined pup tissues (lung, liver, and spleen) for GPCMV viral load, both as a gauge of congenital GPCMV transmission rates and to compare viral loads across vaccine groups (Figure 3). For negative tissues, a viral load of one genome copy per mg of tissue (the limit of the detection of the assay) was assigned for statistical comparisons. Six of 12 pups born to the Venus group (five stillborn and one liveborn pup) had congenital GPCMV infection, as evidenced by a positive PCR signal in one or more tissues. Viral loads of 2539 ± 6885, 11,268 ± 29,350, and 1632 ± 4547 genomes/mg tissue were noted in lung, liver, and spleen from pups from the Venus group, respectively. In the pups born to the gB group, congenital GPCMV transmission was not observed (0/9 pups with vertical transmission; all values at or below the limit of the assay). For the gH/gL group, 1/12 pups (a liveborn pup) was noted to have congenital GPCMV transmission; the calculated mean viral load across pups (using 1 genome/mg as a threshold for detection) was 28.3 ± 98.2 genomes/mg. For the PC group, 1/12 pups (also a liveborn pup) was noted to have congenital GPCMV transmission; again, using the 1 genome/mg threshold determination for negative pups, the calculated mean viral load across pups in this group was 6 ± 18.1 genomes/mg. The differences were statistically significant comparing the Venus control group with each vaccine group for lung viral load and liver viral load (*p* values noted in legend for Figure 3). Thus, vertical CIDMTR transmission occurred in 6/12 pups (50%) in the sham-vaccinated group, compared to 2/34 (6%) in the vaccine groups (*p* = 0.002; one pup included in the mortality comparisons was unavailable for PCR analysis). We conclude that, compared to the control group, that pups born to dams undergoing a second pregnancy following immunization in their first pregnancies with 22122-derived glycoprotein sequences were strongly protected against congenital GPCMV transmission after challenge with a heterologous viral strain variant.

## 4. Discussion

In this study, we build on previous work in the GPCMV model using MVA-vectored vaccines targeting homologs of GPCMV glycoproteins [23]. In a previous study, we demonstrated that vaccines based on GPCMV homologs of (1) gB, (2), gH/gL, and (3) the PC all elicited GPCMV-specific binding and neutralizing antibodies following a three-dose vaccine series in GPCMV-naïve guinea pigs. The vaccines were based on the prototypical 22122 strain of GPCMV [25], and viral challenge during pregnancy took place with SG homogenates of 22122. We found that pre-conception immunization resulted in reductions in maternal DNAemia, improved pups’ birth weights, and reduced mortality and congenital GPCMV transmission, supporting the inclusion of vectored viral glycoprotein complexes in a cCMV vaccine for humans.

We evaluated pregnancy outcomes in dams challenged with salivary gland (SG virus)-adapted 22122 virus, as previously described [25], that had previously been immunized with MVA-vectored subunit glycoprotein vaccines based on strain 22122-specific sequences (vaccines including gB, gH/gL, and the PC). We then asked if the vaccine conferred longer-term protection against reinfection in a second pregnancy, in which dams were now challenged with a heterologous strain of GPCMV, the CIDMTR strain. Since all dams had as similar prior infection with 22122 virus challenge, this served as an internal control for any impact that a wild-type SG virus immune response might have on pup outcomes in a subsequent pregnancy. Previous work with the CIDMTR virus demonstrated that it was capable of causing congenital GPCMV infection in dams previously immune to the 22122 strain. Examination of cross-strain protection conferred by subunit immunization based on a single strain of a virus is a timely and important issue in HCMV vaccines. Mounting evidence indicates that the predominant burden of cCMV infection and disease is conferred by reinfection of pregnant women who have pre-conception immunity to heterologous strains [1,3,6,7,8,15].

Although we were unable to draw any firm conclusions about which of the vectored MVA vaccines provided the greatest degree of efficacy upon challenge with a heterologous strain of virus, clearly prior immunization with an MVA-vectored vaccine construct had a substantial impact on pregnancy outcome compared to placebo (Venus) control. By ANOVA (Figure 1b), the MVA/gB vaccine appeared to demonstrate the greatest impact on maternal viremia following CIDMTR virus challenge. This was unsurprising, since there is little divergence in the gB coding sequence between the 22122 strain and the CIDMTR strain (99% identity at the protein coding level) [21]. The patterns of similarity/divergence between the CIDMTR and 22122 strain (most similar → most divergent) are: gB → gL → GP133 (homolog of HCMV UL131) → GP131 (homolog of HCMV UL130) → GP129 (homolog of HCMV UL128) → gH → gO (Appendix A; Table A1). Future studies will focus on cross-strain protection of glycoproteins for which the most substantial divergence exists. It would be of particular interest to examine variant strain sequences in the context of gB vaccination, since the MVA-gB construct appeared to be the most effective construct in the GPCMV model [23], and since HCMV vaccines have, to date, focused heavily on gB as a protective immunogen [26]. Viruses from infected individuals who were given either the gB/MF59 vaccine or placebo demonstrated that vaccinees were more resistant to infection from viral strains from the gB-1, gB-2, and gB-4 genotype groups, suggesting strain-specific variability in protection following immunization corresponding to a single genotype [26]. These observations have implications for reinfection during pregnancy, as well as the potential limitation of single-genotype vaccines, and these questions can now be modeled with GPCMV.

These studies confirm our previous observations [21] that the CIDMTR strain is able to cause viremia, disease, and congenital GPCMV transmission in animals previously infected with the prototypical 22122 strain. The 22122 strain has a complex history [27,28,29,30]. Between its original isolation by Hartley and its submission to ATCC, it appears to have undergone 54 additional passages in guinea pigs, and six additional passages in cell culture (3 passages in guinea pig embryo fibroblasts, and 3 passages in CCL 158 cells). Inoue and colleagues [30] later demonstrated that the ATCC strain was a mixture of two variants: a full-length variant (GPCMV/full), and a variant designated GPCMV/del, which lacked a 1.6 kb locus that was later determined to encode homologs of the HCMV PC proteins UL128, 130, and 131, which were designated GP129, 131, and 133. Although the ATCC GPCMV 22122 viral stock (http://www.atcc.org/products/all/VR-682.aspx#history/, accessed on: 16 December 2021) contains a 1.6 kb deletion variant, previous work in our lab [31] demonstrated that our salivary gland workpools of virus all contain clonal stock, with no deletion variants.

In this study we demonstrate that the availability of a second, in vivo-passaged “clinical isolate” of GPCMV, the CIDMTR strain, will enhance the ability to model cCMV transmission due to reinfections in the congenital infection model. Reassuringly, since our initial report of the isolation of the CIDMTR isolate, another group has similarly isolated GPCMV from a salivary gland homogenate from a guinea pig obtained from the same commercial vivarium, and has reported findings consistent with our previous observations. This isolate, tentatively designated as TAMYC [32], has been reported to have an unexpected sequence inconsistency in the amino terminus of the GP74 open reading frame (ORF), specifically, codons 32–76, which have no identifiable homologies with any other herpesvirus gO (or other) proteins (Appendix A; Figure A1). Missing from the TAMYC sequence are two glycosylation sites conserved in other gO homologs and, more significantly, the highly conserved tryptophan residue (aa 58, based on the GPCMV strain 22122 coordinates) that is essential for gO function in all betaherpesviruses [33]. This residue, inexplicably, is missing in the TAMYC sequence, which is reported instead to encode for a serine residue at this position. This substitution, if correct, would not be compatible with a viable, replication-competent virus. We note that both the CIDMTR and TAMYC isolates were obtained from the same commercial colony (Charles River Laboratories); both demonstrate epithelial cell tropism; and, except for the aberrant and unexplained sequence spanning codons 32–76 of the GP74, both have identical protein-coding sequences for ORFs gB, gH, gL, GP129, GP131, and GP133. The sequence as reported for codons 32–76 of the TAMYC isolate is, therefore, probably a sequencing mistake. Hence, we conclude, the aberration in codons 32–76 of TAMYC gO notwithstanding, that the CIDMTR and TAMYC strains are, in fact, likely the same virus. This is not surprising given the fact that they were isolated from the same commercial vivarium and have identical sequences. Although small single-nucleotide polymorphisms may be expected following in vivo passage in different animal colonies, they are fundamentally identical viruses. The collection of additional clinical isolates of GPCMV is a high priority area for future work, to best model the properties of maternal reinfection, congenital transmission, and vaccine-mediated protection against cCMV transmission in this uniquely relevant small-animal model of congenital infection.

## 5. Conclusions

In summary, our findings are as follows:

1. The CIDMTR strain of GPCMV, a “clinical isolate” of the virus, is pathogenic in the guinea pig vertical transmission model.
2. Animals immunized with MVA-vectored glycoprotein subunit vaccines based on the prototypical 22122 strain are protected against maternal viremia after challenge with a virulent, in vivo-passaged workpool of CIDMTR virus in a second pregnancy.
3. The magnitude and duration of maternal CIDMTR DNAemia was noted to be statistically reduced in dams previously immunized with MVA/gB vaccine.
4. Upon CIDMTR virus challenge in a second pregnancy, pups born to dams previously vaccinated with 22122 MVA/PC vaccine had statistically enhanced birth weights.
5. All glycoprotein vaccine strategies protected against pup mortality and cCMV transmission in this reinfection model compared to the vector-only control.
6. Based on its isolation from the same commercial vivarium, its biological/in vivo characterization, and its absolute identity at the sequence level for glycoprotein sequences annotated to date, the TAMYC strain of GPCMV is, pending additional sequence analyses/clarification, apparently identical to the CIDMTR strain. This independent confirmation of the novel CIDMTR strain should help inform and direct studies of cross-strain protection engendered by vaccines for HCMV.

## 6. Patents

The CIDMTR strain of GPCMV has been assigned patent number US9395369B2.

## Figures and Tables

**Table 1:** Vaccine Groups Employed for Heterologous Reinfection Study and Previous Pregnancy Outcomes from Initial Vaccine/Pregnancy/Challenge Study.

Vaccine Group	Animals Bred for 2nd Pregnancy	Previous Pup Mortality Post-22122 Challenge	Previous GPCMV Transmission Rate
Venus (Vector)	3	4/4 (100%)	3/3 (100%) ^1^
MVA-gB	2	0/4 (0%)	0/4 (0%)
MVA-gH/gL	3	0/6 (0%)	0/3 (0%) ^1^
MVA-PC	4	0/10 (0%)	0/3 (0%) ^1^
All Vaccine Groups	9	0/20 (0%)	0/10

^1^ Only three pups in these groups were available for evaluation for congenital GPCMV transmission [23]; the mean viral load across all of the pup organs evaluated was 21,609 ± 8722 (SEM) genomes/mg.

**Table 2:** Pup Survival Rates in Vaccinated and Control Dams Following Mid-gestational CIDMTR Challenge.

Vaccine Group	Live Pups	Dead Pups	Mortality Rate
Venus (Vector)	6	6	50%
MVA-gB	9	0	0%
MVA-gH/gL	11	2	15%
MVA-PC	12	1	14%
All Vaccine Groups	32	3	8.6%

**Figure A2:**
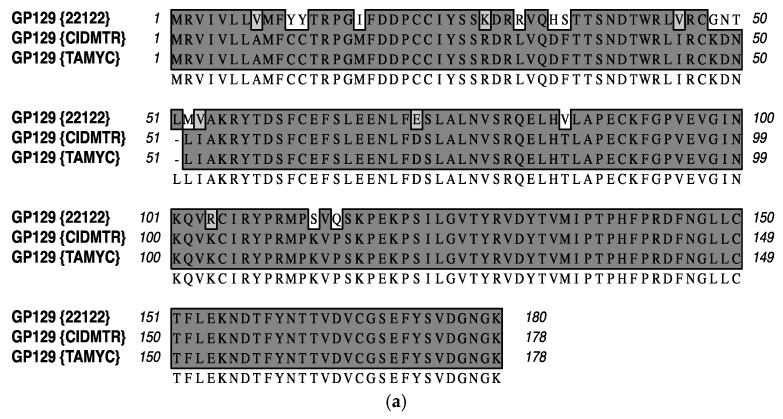

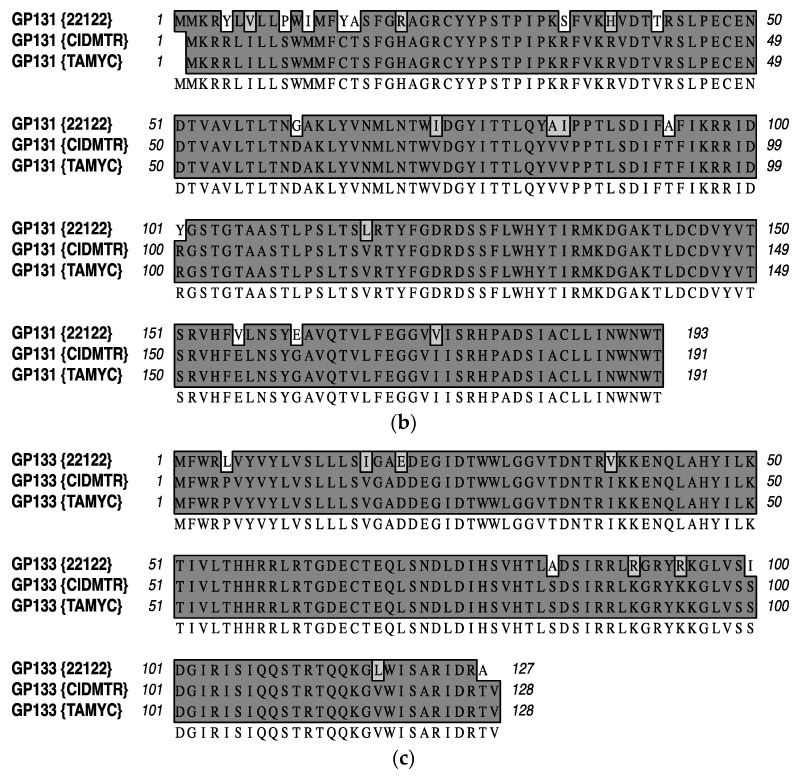
ClustalW comparisons of protein-coding sequences for GPCMV strains 22122, CIDMTR, and the TAMYC strains. Comparisons shown for (**a**) GP129; (**b**) GP131; and (**c**) GP133 all demonstrate 100% identity at the protein-coding level for the CIDMTR and TAMYC isolates, reinforcing that these are likely identical strains independently isolated from the same commercial vivarium.

## Data Availability

Data from this study are stored in a secured site maintained by the UMN Center of Data Excellence (https://it.umn.edu/services-technologies/box-secure-storage, accessed on 16 December 2021). Data are available on request.

## Appendix A

**Table A1:** Glycoprotein coding sequence comparisons: GPCMV strains 22122 and CIDMTR ^1^. The patterns of similarity/divergence between the CIDMTR and 22122 strains (from most similar → most divergent) are as follows: gB → gL → GP133 (homolog of HCMV UL131) → GP131 (homolog of HCMV UL130) → GP129 (homolog of HCMV UL128) → gH → gO.

ORF	Glycoprotein	Percentage Identity
GP55	Glycoprotein B	99.8%
GP115	Glycoprotein L	99.2%
GP133	PC; Homolog of UL131	92.1%
GP131	PC; Homolog of UL130	89.5%
GP75	Glycoprotein H	84.4%
GP74	Glycoprotein O	81.1%

^1^ Based on accession HG531783 (CIDMTR) and KC503762 (22122).
